# Regulatory Effect of Peptidoglycan on the Expression of Toll-Like Receptor 2 mRNA and Proteins in Trophoblast Cell Line TEV-1 Cells

**DOI:** 10.5402/2011/692858

**Published:** 2010-10-07

**Authors:** Yi Wang, Xi-Ping Luo, Chi Eung Danforn Lim, Wu Shun Felix Wong, Gang Zhong

**Affiliations:** ^1^Department of Gynaecology, Guangdong Women and Children's Hospital and Health Institute, Guangzhou, 510010, China; ^2^South Western Sydney Clinical School, Faculty of Medicine, University of New South Wales, P.O. Box 3256, Blakehurst, Sydney NSW 2221, Australia; ^3^Faculty of Medicine, University of New South Wales, Sydney NSW 2052, Australia; ^4^Tongji Hospital, Tongji Medical College, Huazhong University of Science and Technology, Wuhan, 430074, China

## Abstract

*Objective*. To investigate the regulatory effect of peptidoglycan on the expression of human Toll-like receptors 2 (TLR2) mRNA and proteins in the human extravillous trophoblast cell line (TEV-1). *Methods*. TEV-1 cells were incubated with different doses of peptidoglycan. The expression of TLR2 mRNA and protein was detected by reverse transcriptase-polymerase chain reaction (RT-PCR) and immunocytochemistry SP staining. *Results*. TLR2 was expressed in TEV-1 cells and localized to both the cytoplasm and plasma membrane. Compared with the untreated control, TEV-1 cells incubated with 30 *μ*g/ml peptidoglycan significantly upregulated the expression of TLR2 mRNA and protein after 12 hours of treatment (*P* < .01). However, the expression of TLR2 mRNA and protein was decreased but had no significant difference compared with the control (*P* > .05) after 24 hours of treatment. On the other hand, 10 *μ*g/ml peptidoglycan did not seem to have regulatory effect on mRNA and protein expression of TLR2 (*P* > .05). *Conclusion*. Peptidoglycan has a role in regulating the expression of TLR2 mRNA and protein in TEV-1 cells. It suggests that the trophoblast cells may play important role in the immune response at the fetal-maternal interface and affect the result of pregnancy by expressing TLR2.

## 1. Introduction

The maternal-fetal interface represents an immunologically unique site to protect fetus by maintaining host defense against a diverse array of possible pathogens. As a consequence, either an inefficient clearance of an infectious agent or an overactive immune response may have a significant impact on the pregnancy. Clinical studies have shown a strong association between certain complications of pregnancy and intrauterine infections [1–3]. It is reasonable to believe that innate immune responses against microorganisms at the maternal-fetal interface may have a significant impact on the success of a pregnancy. 

The innate immune system responds to infection through a system of pattern recognition receptors (PRRs) which recognize conserved sequences known as pathogen-associated molecular patterns (PAMPs), such as bacterial lipoproteins, LPS, and peptidoglycan (PGN) [4]. One of the main families of PRR is toll-like receptors (TLRs). TLRs are members of a larger family of proteins that includes the interleukin 1 (IL1) receptor and share a conserved cytoplasmic domain called the Toll/IL1R (TIR) domain. TLR signals through the adapter molecule, MyD88, to activate the NF-*κ*B pathway, which results in an immune response characterized by the production of cytokines, antimicrobial products, and the regulation of costimulatory molecules [5, 6]. Currently there are 13 mammalian Toll-like receptors homologues that have been identified—TLR 1–13. In humans, ten functional TLRs (TLRs 1–10) have been identified [7, 8]. And studies have demonstrated the expression of TLR1-10 mRNA in human term placenta [9, 10]. Among the TLRs, TLR2 is believed to recognize most various infectious pathogens including peptidoglycan (PGN), which is the main component of the cell wall in Gram-positive bacteria. 

The immunological microenvironment at the maternal-fetal interface consists of the trophoblast cells, immunological cells, and the cytokines [11, 12]. There is growing evidence that trophoblast cells are able to recognize and respond to pathogens through the expression of TLRs. The extravillous trophoblasts are capable of invading the maternal decidualized endometrium and being part of the fetal cells which have the direct contact with maternal immunological system. TEV-1 cell line was originally obtained from primary cultures of human first-trimester placenta which retains all the established criteria for extravillous trophoblasts [13]. 

It is proposed that the human extravillous trophoblasts at the maternal-fetal interface can recognize and respond to pathogens through expressing TLRs, which may ultimately affect the outcome of pregnancy. In order to study the role of TLR2 at maternal-fetal interface, TEV-1 cell was used in this project as an in vitro model. The regulation of TLR2 by microbial stimulus was assessed by incubating TEV-1 cells with PGN and analysed with reverse transcriptase-polymerase chain reaction and immunocytochemistry.

## 2. Materials and Methods

### 2.1. Materials and Reagents

The TLR2 active ligand—a highly purified S. aureus peptidoglycan—was used in this project, and it was obtained from FLUKA (now Sigma, USA). The mouse monoclonal anti-TLR2 antibody (Santa,USA), charcoal-stripped FBS (Gibco, USA), SP kit (Wuhan Boster, China), and TRIzol (Invitrogen, USA) were used in the experiments. Primers (Shanghai Boya, China), RNAsin, dNTP, M-MLV, oligo dT Primer, and Taq enzyme (Promega, USA) were utilized in this project.

### 2.2. Cell Culture

The human extravillous trophoblast cell line TEV-1 cells were cultured in DMEM/F12 without phenol red, supplemented with 10% FBS, 12 mM L-glutamine, and 100 nm of penicillin/streptomycin. The human extravillous trophoblast cell line TEV-1 cells (1 × 106/ml) were then incubated with no treatment (medium) as a negative control, with 10 *μ*g/ml PGN for 12 hours or 24 hours, or with 30 *μ*g/ml PGN for 12 hours or 24 hours.

### 2.3. Reverse Transcription-Polymerase Chain Reaction (RT-PCR) Analysis

Total RNA was isolated from the human extravillous trophoblast cell line TEV-1 cells by the Trizol method according to the manufacturer's instructions. Two micrograms of total RNA reversely transcribed using dT15 oligonucleotide and Moloney murine leukemia virus reverse transcriptase in a 20 *μ*l total reaction volume. Two microliters of the reverse transcriptase reaction subjected to PCR analysis to measure the mRNA levels of TLR2 and *β*-actin. PCR amplification was performed for TLR2 and *β*-actin with Taq polymerase for thirty cycles at 94°C for thirty seconds, 54°C for forty seconds, and 72°C for one minute. Primers for TLR2 were 5′-GATGCCTACTGGGTGGAGAA-3′ sense and 5′-CGCAGCTCTCAGATTTACCC-3′ antisense while the size of the product was 393 bp. PCR primers for *β*-actin were 5′-AGGCCGGTGCTGAGTATGTC-3′ sense and 5′-TGCCTGCTTCACCACCTTCT-3′ antisense, and the size of the product was 530 bp. The PCR products were separated by electrophoresis in a 2% agarose gel at 75 volts followed by staining with 1 : 20 000 dilution of Goldview (a kind of nucleic acid dye). A 600 bp ladder was included with all experiments to confirm the expected band size. The bands were then visualized by ultraviolet transillumination and measured on the basis of the English gel image analysis system GDS-8000. The intensity of each band was then normalized to its corresponding *β*-actin band to semiquantitatively compare the values between samples.

### 2.4. Immunocytochemistry

TEV-1 cells were harvested 12 or 24 hours after stimulation, and they were fixed with ice acetone for twenty minutes followed by being washed three times with PBS. The preparations were incubated in a 3% H_2_O_2_ for ten minutes in order to quench endogenous peroxidase activity. Paraformaldehyde-fixed trophoblast cells, previously adhered to glass slides, were blocked with 5% goat serum in PBS for twenty minutes at room temperature. Following three washes with PBS, samples were incubated overnight at 4°C with the anti-TLR2 monoantibodies while the PBS was served as negative controls. In addition, specific staining after three washes with PBS was also detected by incubating with a peroxidase-conjugated goat antimouse Ab (1/100 dilution) for thirty minutes followed by a five-minute incubation with diaminobenzidine substrate (Vector Laboratories). Cells and tissue sections were then counterstained with hematoxylin (Sigma-Aldrich) before dehydration with ethanol. Slides were visualized by light microscopy, and the HPIAS-2000 computerized image analysis system was used to quantitatively determine the expression of TLR2 protein in terms of staining intensity. Three randomly selected fields were obtained from each slide to obtain a mean value (OD value—a mean value of immunostaining intensity).

### 2.5. Statistical Analysis

All experiments were repeated three times, and data were presented with mean ± standard deviation. Statistical analyses were carried out by using the SPSS12.0 software, and one-way ANOVA was also used for statistical purpose. Result with *P* < .05 was considered to be statistically significant in this experiment.

## 3. Results

### 3.1. The Human Extravillous Trophoblast Cell Line TEV-1 Cells Express TLR2

The first objective of this study was to determine the expression pattern of TLR2 in the human extravillous trophoblast cell line TEV-1 cells. As shown in Figure 2(a), TEV-1 cells showed positive immunoreactivity for TLR2, and the positive brown granules were mainly distributed in the cytoplasm and cell membrane. That means that TLR2 was localized both on the cell surface as well as intracellularly in TEV-1 cells.

### 3.2. TLR2 mRNA and Protein Expression after PGN Stimulation

**Figure 1:**
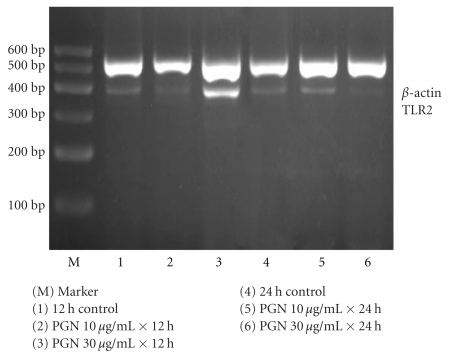
The expression of TLR2 mRNA in TEV-1 cells.

**Figure 2:**
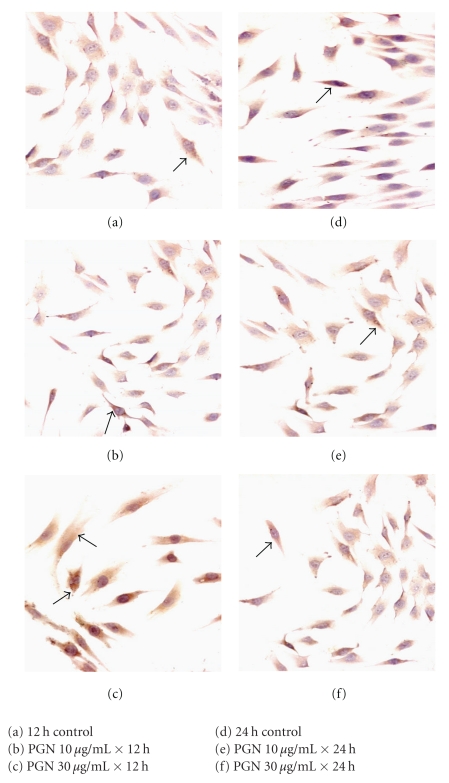
The expression of TLR2 protein in TEV-1 cells (immunocytochemistry SP × 200).

As shown in Figures 1 and 2, as well as Table 1, TEV-1 cells were stimulated with different concentrations of PGN or incubated with medium alone for 12- and 24-hour period. Compared with the control, TEV-1 cells incubated with 30 *μ*g/ml peptidogly can be shown to significantly upregulate the expression of TLR2 mRNA and protein after 12 hours of treatment (*P* < .01). However, when it was allowed to last to 24 hours, the expression of TLR2 mRNA and protein were decreased (as compared with the 12-hour group) but had no significant difference compared with the control (*P* > .05). On the other hand, 10 *μ*g/ml peptidoglycan did not affect the expression of TLR2 mRNA and protein after either 12 hours or 24 hours (*P* > .05) of treatment.

## 4. Discussion

Intrauterine infection affects the placental development and function. It can subsequently leads to complications of pregnancy such as preterm labor and delivery, fetal growth restriction (FGR) and preeclampsia [1–3]. However, the precise adverse reaction of intrauterine infection is yet to be defined. TLRs are the key components of the innate immune system which recognize conserved sequences on the surface of pathogens and trigger the effector cell functions. Researchers found that TLR2 expression was significantly higher in patients with chorioamnionitis than in those without this condition [14]. The TLR2 expression was found to be polarized to the basal surface of amniotic epithelial cells in patients without chorioamnionitis, but this distribution was lost in the presence of inflammation. It is suggested that TLR2 may play an important role in immunological respondse at maternal-fetal interface [14]. In our study, immuno-cytochemical staining of TEV-1 cells revealed that the TLR2 was highly expressed intracellularly as well as being expressed on the cell surface. Such cytoplasmic expression may provide rapid mobilization of additional receptors to the cell surface following initial bacterial recognition. Alternatively, cytoplasmic expression may serve to facilitate intracellular recognition and responses. The cytoplasmic expression was also found in human dendritic cells [15].

A study in the human placenta cultures demonstrated that stimulation with zymosan and LPS for 2–6 hours readily induced interleukin- (IL- ) 6 and IL-8 cytokine production, whereas TLR2 mRNA and protein expression remained at the same high level as in unstimulated explants [16]. However, in our experiments we found that, when comparing with the untreated control, TEV-1 cells incubated with 30 *μ*g/ml PGN could significantly up-regulate the expression of TLR2 mRNA and protein after 12 hours of treatment (*P* < .05). When the incubation period lasted to 24 hours, the expression of TLR2 mRNA and protein was decreased as compared with the 12 hours group, even though there was no statistically significance while comparing with the control (*P* > .05). It is proposed that the difference of the results obtained in our experiments and other reported studies may be related to different kinds and doses of stimulants used, different cells and tissue applied, and also the duration of stimulation applied. 

In our experiment, two time points 0–12 hours–24 hours and two dose points 0–10 *μ*g/ml–30 *μ*g/ml were set for determination of outcomes. In the 12 hours of treatment group, 10 *μ*g/ml PGN did not affect the expression of TLR2 whereas 30 *μ*g/ml PGN increased the expression. This suggested that there may be a dose-dependent manner, which may conduce to recognize and respond to more pathogens. In the 24 hours treatment group, 10 *μ*g/ml PGN still did not affect the expression of TLR2, but 30 *μ*g/ml decreased the expression. We believed that when stimulate time is prolonged, the dose-dependent manner may disappear due to the high dose PGN-induced cells apoptosis. There are reports on PGN to induce trophoblast apoptosis through TLR2 [17]. Holmlund et al. (2002) [16] incubated the first trimester trophoblast cells (H8) with PGN (80 *μ*g/ml), following 48 hours of treatment. It resulted in a 61.72% increase in the number of apoptotic trophoblast cells compared with the untreated control. It was suggested not related to simple dose- and time-dependent manner but rather a complicated regulatory effect. Thus the extravillous trophoblast cells may play important roles in the immune response at the fetal-maternal interface.

## 5. Conclusion

The mRNA and protein of TLR2 are shown to express in the human extravillous trophoblast cells TEV-1, and peptidoglycan may stimulate the expression of TLR2 in TEV-1 cells. It is indicated that the extravillous trophoblast cells may play important roles in the immune response at the fetal-maternal interface and affect the pregnancy outcome by expressing TLR2. Such altered trophoblast cell responses may contribute to the pathogenesis of certain pregnancy complications.

## Figures and Tables

**Table 1:** The expression of TLR2 in TEV-1 cells ($\overset{®}{x}\pm s$).

Groups	TLR2 mRNA	TLR2 protein
12 hours		
Control	0.212 ± 0.028	0.135 ± 0.016
PGN 10 *μ*g/ml × 12 hours	0.261 ± 0.059	0.135 ± 0.021
PGN 30 *μ*g/ml × 12 hours	0.415 ± 0.063*	0.200 ± 0.025*
24 hours		
Control	0.200 ± 0.016	0.132 ± 0.016
PGN 10 *μ*g/ml × 24 hours	0.198 ± 0.024	0.134 ± 0.019
PGN 30 *μ*g/ml × 24 hours	0.145 ± 0.014	0.122 ± 0.014

*P < .01 as compared with the other five groups.
